# Towards an integrative structural biology approach: combining Cryo-TEM, X-ray crystallography, and NMR

**DOI:** 10.1007/s10969-014-9179-9

**Published:** 2014-04-20

**Authors:** Jeffrey Lengyel, Eric Hnath, Marc Storms, Thomas Wohlfarth

**Affiliations:** FEI Company, 5350 N.E. Dawson Creek Drive, Hillsboro, OR 97124 USA

**Keywords:** Cryo-electron microscopy, Cryo-transmission electron microscopy, Structural biology, Hybrid methods

## Abstract

Cryo-transmission electron microscopy (Cryo-TEM) and particularly single particle analysis is rapidly becoming the premier method for determining the three-dimensional structure of protein complexes, and viruses. In the last several years there have been dramatic technological improvements in Cryo-TEM, such as advancements in automation and use of improved detectors, as well as improved image processing techniques. While Cryo-TEM was once thought of as a low resolution structural technique, the method is currently capable of generating nearly atomic resolution structures on a routine basis. Moreover, the combination of Cryo-TEM and other methods such as X-ray crystallography, nuclear magnetic resonance spectroscopy, and molecular dynamics modeling are allowing researchers to address scientific questions previously thought intractable. Future technological developments are widely believed to further enhance the method and it is not inconceivable that Cryo-TEM could become as routine as X-ray crystallography for protein structure determination.

Cryo-transmission electron microscopy (Cryo-TEM), and particularly single particle analysis, is becoming an essential technique for structure determination of viruses and protein complexes. Single particle analysis is a structural technique where 2D transmission electron micrographs of individual, randomly orientated protein complexes or viruses can be mathematically aligned, through image processing techniques, to generate a 3D volume of the specimen [1–5]. The samples are typically either encased in a heavy metal stain (negative stain), such as uranyl acetate, or imaged at cryogenic temperatures with the sample embedded in vitreous ice, free of strain (Cryo-TEM) [6, 7]. In order to prepare specimens for Cryo-TEM, the specimen is applied to a carbon coated EM grid with a series of small holes (µm size range). The sample is blotted away, leaving a thin film with the specimen residing within the small holes. The grid is then rapidly plunged into liquid ethane or propane and cooled to liquid nitrogen temperatures. The low vapor pressure and high thermal capacity of the liquid ethane or propane results in extremely rapid freezing where ice crystals are not formed, rather water molecules are trapped in the same orientation they had in the aqueous phase. This forms amorphous, vitreous ice and preserves the specimen in a frozen hydrated state, which mirrors the native state. The specimen must then be maintained at cryogenic temperatures in order to prevent a phase transition, which would result in formation of crystalline ice and damage to the specimen [7]. The resulting sample is subsequently imaged in an electron microscope, and reconstruction software is used to create the 3D structure from the individual particle images. Although individual particles are imaged at low contrast, the resulting structure can be extremely high resolution due to averaging of thousands of particles.

3D electron density maps are routinely deposited in the electron microscopy database (EMDB) www.emdatabank.org. “The EMDataBank is a unified global portal for deposition and retrieval of 3DEM density maps, atomic models, and associated metadata, as well as a resource for news, events, software tools, data standards, validation methods for the 3DEM community” [8]. Furthermore atomic coordinates fitted into TEM density maps are deposited in the protein databank (PDB). http://www.rcsb.org/pdb/home/home.do. “The PDB archive is a repository of atomic coordinates and other information describing proteins and other important biological macromolecules.” Both the Cryo-TEM density maps and fitted atomics coordinates can be freely downloaded from these websites.

Classically single particle analysis, particularly when applied to Cryo-TEM studies, has been thought of as a very low resolution (~30 Å resolution) structural technique that can be applied to only very rigid, megadalton sized protein complexes; such as the ribosome, or highly symmetric icosahedral viruses. While large protein complexes and viruses are still being studied, Cryo-TEM has undergone a revolution, aided by advancements in sample preparation, electron microscope technology, automation in data collection, detector technology, and image processing [2–5]. Taken as a whole, these advancements have allowed Cryo-TEM researchers to determine protein structures at atomic resolution and addressed biological research questions previously thought unattainable by the method. For instance in 2009 Zhang et al. [9] published the seminal atomic resolution 3.3 Å aquareovirus structure in the journal Cell where individual side chains were clearly resolved (Fig. 1) (EMDB Accession code: EMD-5160; Fitted PDB ID: 3IYL). Due to the extremely high resolution obtained in the structure, autolytic cleavages site were visualized which revealed a novel priming mechanism for cell entry for non-enveloped viruses. The same year Liu et al. [10] published a 3.6 Å structure of adenovirus in the journal Science (EMDB Accession code: EMD-5172; Fitted PDB ID: 3IYN). In the same issue, an X-ray crystal structure of adenovirus at 3.5 Å was also published [11] (PDB ID: 1VSZ). Steven Harrison (Harvard) wrote in a commentary about the two structures, stating that while the X-ray structure was at higher resolution, the Cryo-TEM structure actually showed substantially better details on a more difficult and more biologically relevant type of the virus [12]. Cryo-TEM had unequivocally bested X-ray crystallography when applied to viruses and is currently considered the premier method for icosahedral virus structure determination. This is further echoed in a recent publication (confessions of an icosahedral virus crystallographer) by structural virologist, Jack Johnson (Scripps Research Institute), where he details his methodological change from using X-ray crystallography to study virus structure to solely using Cryo-TEM [13].

**Fig. 1:**
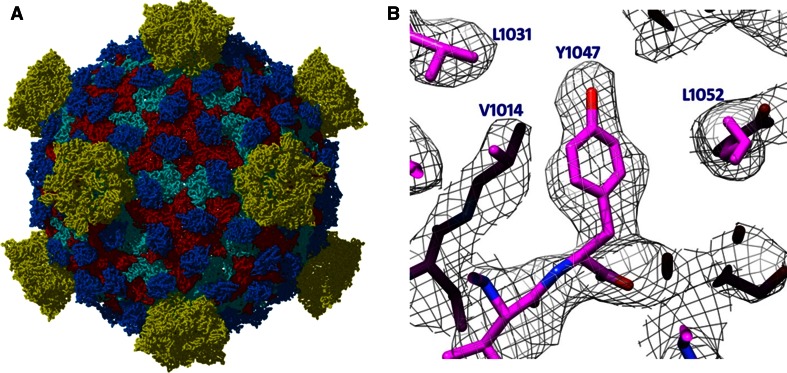
**a** 3.3 Å structure of a non-enveloped icosahedral virus using Cryo-TEM. **b** Image of a selected region were individual side chains are clearly visible in the Cryo-TEM reconstruction validating the high resolution of the reconstruction. Images kindly provided by Z. Hong Zhou Electron Imaging Center for Nanomachines (EICN) CNSI and Department of Microbiology, Immun & Mol. Genetics, UCLA, and adapted from [9]. EMDB Accession code: EMD-5160; Fitted PDB ID: 3IYL

Cryo-TEM is also rapidly being applied to smaller (sub-megadalton) protein complexes. For instance Cryo-TEM has been proven the superior method for studying the trimeric HIV surface spike and has been instrumental in proving that the spike can undergo dramatic conformational changes upon binding to the target host cell proteins [14–16] (Fig. 2) (EMDB Accession codes: EMD-5462; Fitted PDB ID: 3IYN). Visualization of these conformational states could prove critical in future drug design. Currently, numerous research groups are now applying Cryo-TEM methods to sub 500 kDa sized proteins complexes, and recently Wu et al. [17] published a sub-nanometer structure of HIV integrase bound to Fabs resulting in a total molecular weight of 110 kDa (EMDB Accession code: EMD-5294).

**Fig. 2:**
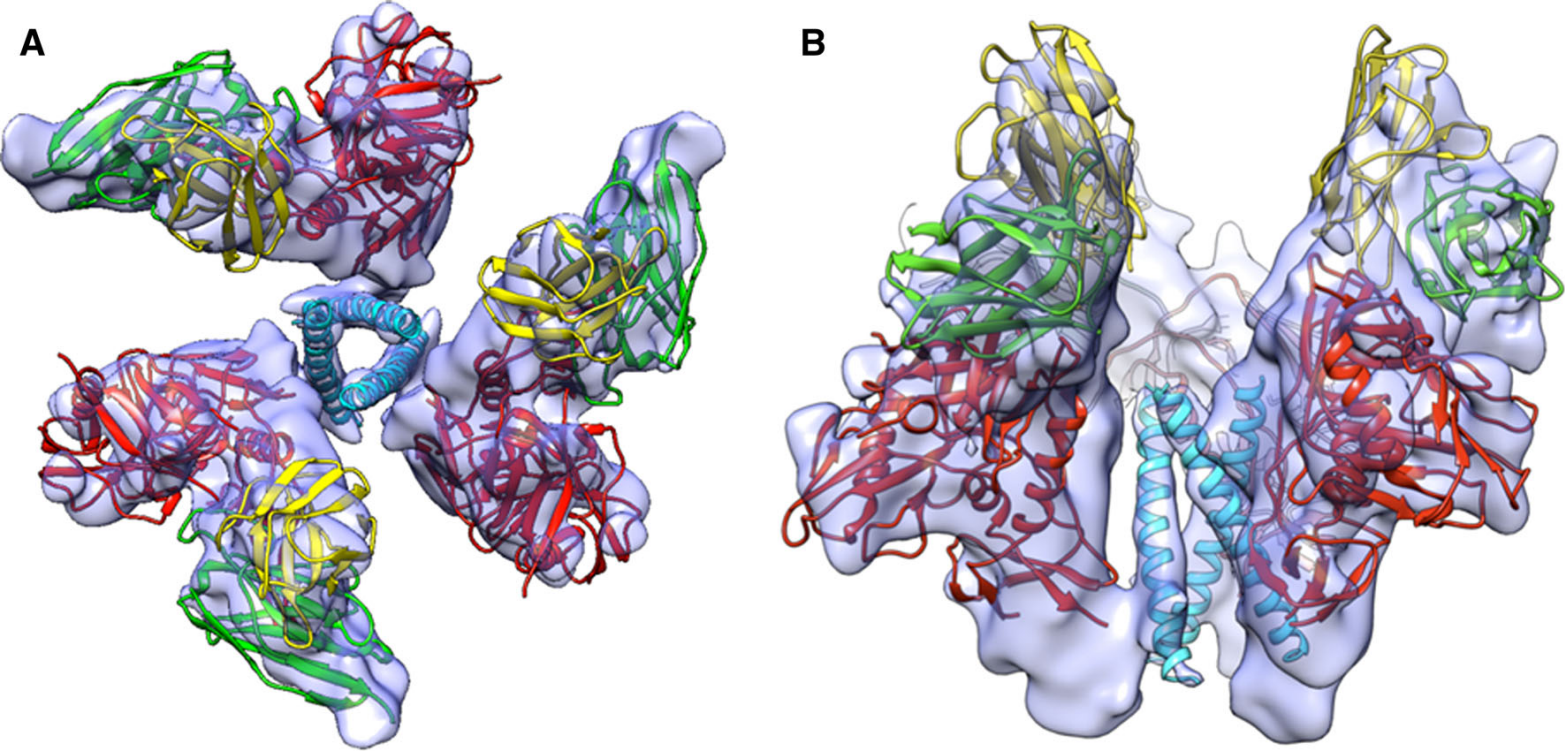
9 Å three-dimensional reconstruction of soluble gp140 HIV envelope glycoprotein trimers bound to three copies of the Fab fragment from 17b, a neutralizing antibody whose binding mimics that of the co-receptor. The structure revealed the presence of a previously unknown “activated” intermediate state, where three buried helices become exposed and potentially accessible to binding by entry inhibitors. **a** Top view of the trimer and **b** Side view of the trimer. Images kindly provided by Sriram Subramaniam, Lab of Cell Biology, National Cancer Institute, National Institutes of Health. Adapted from [16]. EMDB Accession code: EMD-5462

One of the more common biological systems applied to Cryo-TEM research is the ribosome. The ribosome is often used as a standard test specimen to assess the validity of Cryo-TEM technological improvements. While some of the very early ribosome structures solved by single particle analysis were at very modest resolutions <50 Å, currently researchers are able to routinely solve ribosome structures at nearly atomic resolution. For instance, Bai et al. [18] developed a rapid data collection and image processing scheme where, within a week, a ~4 Å ribosome structure can be solved (EMDB Accession codes: EMD-2275, EMD-2276, EMD-2277). The process is so rapid that it is considered “in silico purification” which can assess the presence bound co-factors and ribosomal accessory proteins. Recent advancements in the use of complementary metal–oxide–semiconductor (CMOS) direct electron based detectors to record images have been essential to these methods.

Not only has Cryo-TEM been able to generate very high resolution reconstructions of the ribosome, it has proved invaluable to visualizing dramatic structural changes that occur during RNA translation. Using “4D” or time resolved Cryo-TEM, Fischer et al. [19] have been able to reconstruct numerous intermediate structural states of ribosome within a single dataset using advanced image processing algorithms (Fig. 3). Combining these structural intermediates resulted in a movie of the process of translation. Additionally, by determining the occupancy of each conformational state it was possible to calculate the entire thermodynamic landscape through the whole dynamic process. In total Cryo-TEM has resulted in numerous insights into ribosome biology. For instance Hashem et al. [20] have been able to show how viral messenger RNA can displace eukaryotic initiation factors in order to promote translation of viral mRNA (EMDB Accession codes: EMD-2450, EMD-2451; Fitted PDB ID: 4C4Q).

**Fig. 3:**
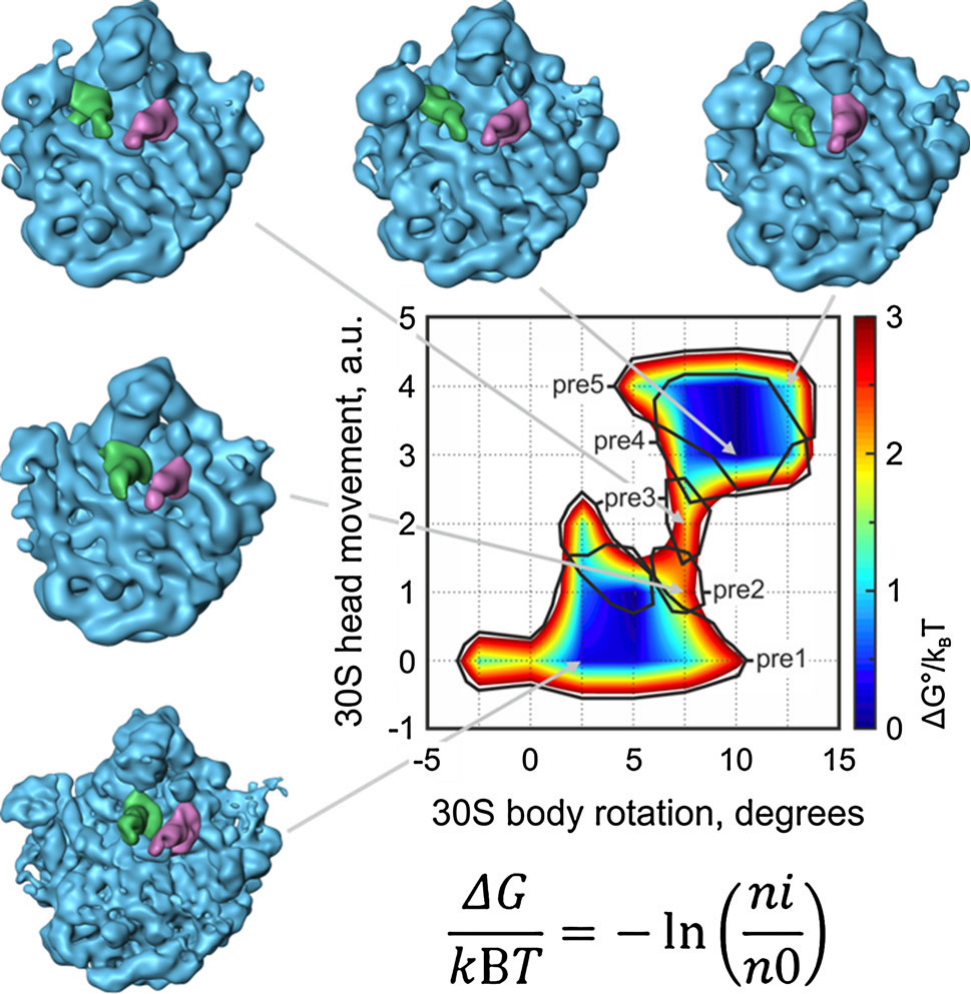
Use of time-resolved Cryo-TEM to visualize ribosome dynamics and tRNA movement. Furthermore, using the ratio of particles residing in each state, the entire thermodynamic dynamic landscape of the transition between states was determined. This is the first example of how using Cryo-TEM structural information to calculate thermodynamic parameters. Image kindly provided by Niels Fischer and Holger Stark, MPI Gottingen. Adapted from [19]. EMDB Accession codes: EMD-1716, EMD-1717, EMD-1718, EMD-1719, EMD-1720, EMD-1721, EMD-1722, EMD-1723, EMD-1724, EMD-1725, EMD-1726, EMD-1727; Fitted PDB IDs: 3J4V, 3J52, 3J4Z, 3J50, 3J4Y, 3J51, 3J53, 3J54, 3J57, 3J58, 3J59, 3J5A, 3J5B, 3J5C, 3J5H, 3J5J, 3J5J, 3J5K

Single particle analysis has resulted in numerous biological insights, however as is the case with several other research methods, it is the combined use of various techniques that often result in the most significant scientific advancements, where the whole is greater than the sum of its individual parts. There is strong belief that hybrid methods are the future of structural biology, with Cryo-TEM being an essential method in these studies. In this context, two studies in particular, combining Cryo-TEM, X-ray crystallography, and molecular dynamics simulations have resulted in groundbreaking results. For instance, a recent publication combined Cryo-TEM structural studies of the areolysin pore with X-ray crystallography and molecular dynamics analysis [21]. Areolysin is a member of an important and widely distributed pore forming protein. There were several essential questions in the pore forming field, such as how areolysin like proteins oligomerize and form pores in the membrane. To address these questions Degiacomi et al., used Cryo-TEM single particle analysis to generate 3D reconstructions in three intermediate states ranging from a heptameric prepore state to a transition to the pore state (Fig. 4).

**Fig. 4:**
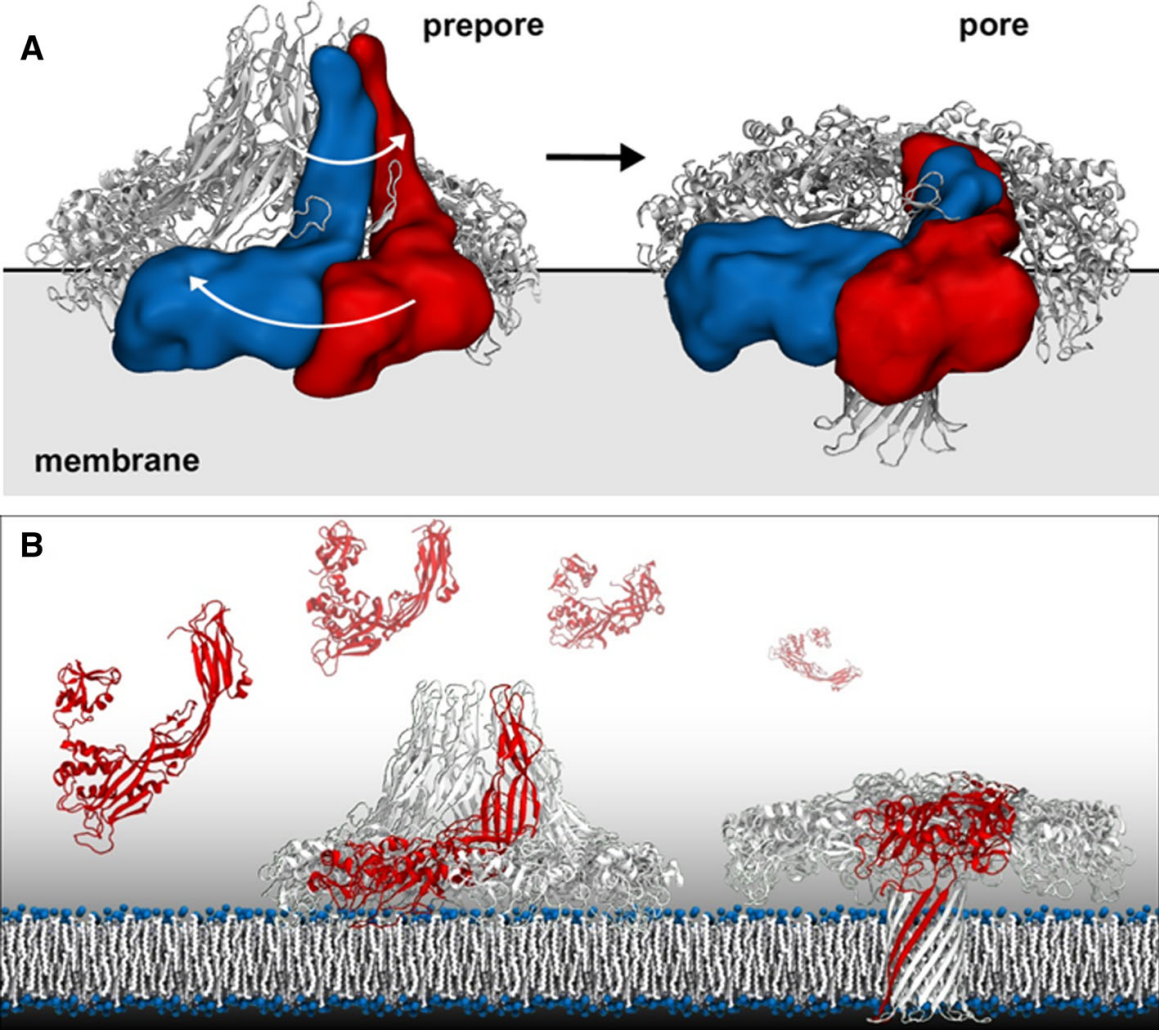
Cryo-TEM reveals various conformational states of areolysin pore formation where a prepore state transitions to a functional toxin pore inserted in the target membrane (**a**). Combining X-ray crystallography, Cryo-TEM, and molecular dynamics modeling revealed a novel swirling membrane insertion mechanism to form the pore, allowing for an atomic resolution interpretation of the transition from monomer to prepore to the functional pore state (**b**). Images kindly provided by Matteo Dal Peraro, Biomolecular Modeling—LBM Institute of Bioengineering, School of Life Sciences École Polytechnique Fédérale de Lausanne—EPFL & Swiss Institute of Bioinformatics—SIB Adapted from [20]

The monomeric crystal structure, in combination with earlier EM analysis, resulted in a perplexing issue.  Thesugar moieties present in glycosylphospatidylinositol-anchored proteins face away from the membrane, as well as having receptor binding sites positioned 10 nm above the membrane surface. Both of these observations were in direct conflict with biochemical information. Cryo-TEM provided elegant clarity to this issue as well as a mechanism for pore insertion into the membrane. Visualization of the prepore states and intermediate states showed an unexpected and dramatic conformational change due to a major twisting motion where areolysin converts the prepore state into the pore state by folding inside out. This allows the pore to insert into the membrane in the functional pore state. When combined with the crystal structure and molecular dynamic simulation this entire process was modeled and understood at the atomic scale and resulting in a very satisfying correlation between biochemical information and various structural information. This study is truly an excellent example of the synergy of hybrid methods resulting in a major breakthrough in this field of research. Moreover, while the single particle reconstructions were solved at modest resolution (16–18 Å), they independently yielded an incredible amount of biochemical insight.

Similar to the aerolysin research, hybrid methods were applied to analysis of HIV capsid formation [22]. Zhao et al. applied specialized Cryo-TEM methods to address capsid formation: helical analysis and cryo-electron tomography (CET). Whereas single particle analysis is typically applied to monodispersed protein complexes randomly oriented in vitreous ice; Helical analysis is applied to periodic helical or tubular structures. While there are several examples of helical filaments in biology, it is also possible to induce proteins to form 2D periodic sheets or tubes [23]. Zhao et al. was able to form tubular arrays of hexameric capsid proteins and generate an 8 Å reconstruction of the capsid protein. While the X-ray crystal structure of the capsid protein was previously solved, the tubular reconstruction revealed novel contact sites between capsid pentamers that are essential in supporting the formation of the full viral capsid. These same contacts supported formation of the periodic tube. After docking of the X-ray crystal structure into the Cryo-TEM density map an all atom molecular dynamic simulation was performed. Without the novel contact site discovered by Cryo-TEM, it would not have been possible to perform the simulation.

In the same publication the structure of the mature viral capsid was also determined. The HIV viral capsid forms a fullerene cone comprised of pentameric and hexameric capsid proteins. A fullerene cone requires 12 pentamers inserted into a hexagonal lattice to close the ovoid. However, there are several possible positions where pentamers can be inserted into the hexagonal lattice allowing for formation of the cone structure. This results in multiple forms of fullerene cones. Just as predicted by Euclidian geometry of fullerenes, HIV viral capsids also visually exhibit this expected variation in shape and geometry [24]. Since the HIV capsid it is a highly variable, pleomorphic structure and is therefore intractable to structural techniques that require averaging of structurally homogeneous particles. However CET is ideally suited to study such structures. CET is analogous to a CT scan, where a scanner rotates around a specimen (i.e. a person), collecting images at each tilt allowing for generation of a 3D reconstruction of the specimen. In CET, instead of tilting the microscope, the specimen is tilted within the column of the electron microscope [4, 25–27]. With this method 3D tomographic volumes of individual mature capsids were generated. Combining a lower resolution tomographic volume as a constraint, the structures of pentamers, hexamers, and novel contact sites determined by tubular analysis, allowed for creation of an atomic model of the entire HIV capsid and subsequently a whole atom molecular dynamic simulation of the entire capid as well (Fig. 5) (EMDB Accession codes: EMD-5582, EMD-5639; Fitted PDB IDs: 3J34, 3J4F, 3J3Y). To date it is the largest molecular dynamics simulation on a protein and was truly a heroic effort. More importantly the model highlighted the threefold capsid protein C-terminal domain as an attractive therapeutic target and provided extremely useful biochemical information.

**Fig. 5:**
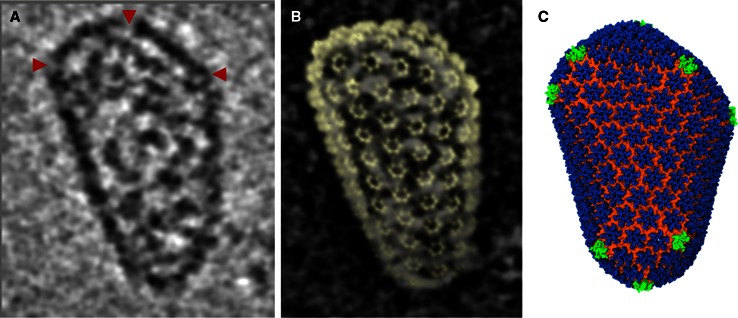
Using cryo-tomographic analysis of individual, native “fullerene” cone HIV-1 capsid, combined with the high resolution Cryo-TEM hexamer structures an all-atom molecular dynamics HIV-1 capsid model was created. This model highlights the threefold capsid protein C-terminal domain as an attractive therapeutic target. **a** Slice through cryo-tomographic volume of an individual mature capsid. *Red arrows* highlight CA pentamers. **b** The tomographic density matches the shape and size of the capsid, shown by the overlay of densities from the segmented capsid and the fullerene model (*yellow*). **c** Final molecular dynamics equilibrated all-atom capsid model. Images kindly provided by Peijun Zhang Univ. of Pittsburgh. Adapted from [22]. EMDB Accession codes: EMD-5582, EMD-5639; Fitted PDB IDs: 3J34, 3J4F, 3J3Y

While Cryo-TEM and X-ray crystallography have been classically very complementary and mutually beneficial techniques, the combination of Cryo-TEM and nuclear magnetic resonance (NMR) spectroscopy is also gaining traction as a highly useful integrated structural technique. Just as atomic coordinates from X-ray crystal structures are commonly docked into Cryo-TEM density maps, atomic coordinates from 3D NMR solution structures can also be docked into Cryo-TEM density maps. Moreover NMR also has the added advantage of studying flexible and dynamic regions within biological specimens. For instance Frye et al. elegantly combined Cryo-TEM and NMR. Not only did the study generate a 3D single particle structure of the anaphase-promoting complex/cyclosome (APC/C), but also simultaneously showed by NMR that intrinsically disordered D-box, linker, and tail elements within the complex are critical for inhibition of activity. These disordered regions were localized in the Cryo-TEM density map by generating creating multiple single particle reconstructions of mutants APC’s with insertions of novel domains near the disordered regions. The new regions of density, due to the added domains, served as markers for the disorder regions [28] (EMDB Accession code: EMD-2353, EMD-2354. This study is represents a true hybrid approach again showing the synergy of using multiple structural methodologies. The analysis was further supported by additional biophysical techniques like dynamic light scattering (DLS), and analytical ultra-centrifugation. In the same publication, NMR was also used to solve the solution structure of a domain of the APC complex.

In addition to the Zhang lab applying helical analysis and CET to study HIV capsids [20], they also combined helical analysis and NMR spectroscopy, in collaboration with the Gronenborn lab, to the study of HIV capsids. Byeon et al. generated a 3D structure of a helical array of hexameric capsid proteins as well as the NMR solution structure of the C-terminal domain (CTD) of capsid proteins. Docking the CTD solution structure into the helical Cryo-TEM density map revealed novel intersubunit interactions between interfaces of the CTDs. These interactions were not previously seen in X-ray crystallographic studies [29] (EMDB Accession code: EMD-5136; Fitted PDB ID: 2KOD). These multiple and distinct intermolecular interactions among the CTD’s provide the necessary plasticity to allow for controlled virus capsid disassembly and assembly. These adjustable and modular interfaces could also potentially explain how generic lattices of hexameric capsid proteins have the necessary flexibility to form variable fullerene cones structures upon addition of 12 pentameric capsid proteins. Once again the merging of multiple structural techniques resulted in novel scientific findings.

Integrating multiple structural methodologies is becoming a common and standard practice for all areas of structural biology research. For instance small angle X-ray scattering (SAXS) data is being used as a constraint to aid in solving solid state and solution NMR structures of “large” (>20 kDa) biological samples Grishaev et al. [30]. However, like X-ray crystallography, both NMR and SAXS studies require a fully homogeneous sample free of aggregation or contamination. Cryo-TEM is unique among structural techniques in that is has single molecule resolution, where individual particles are picked from a field of view. As such sample requirements for Cryo-TEM can be less stringent compared to other structural techniques. With that said, if samples are of sufficient quality for X-ray crystallographic or NMR studies, they are typically suitable for Cryo-TEM. Furthermore as Cryo-TEM is rapidly approaching atomic resolution, it is entirely feasible to consider that integrated structural techniques utilizing Cryo-TEM with X-ray crystallography and/or NMR will be the leading structural approach in the near future. Particularly, as Cryo-TEM can be applied to structural studies of low occupancy and dynamic proteins complexes considered intractable to analysis by other structural techniques.

It is becoming abundantly clear that Cryo-TEM is an essential research technique in structural biology, whose application and functionality will only grow into the future. The pace of research and number of publications are increasing rapidly each year. And more importantly the complexity and variation of experiments is increasing as well. Several studies currently undertaken by Cryo-TEM researchers would not have been considered possible even a year or two ago. The use of robotics in sample loading, improvements in TEM optical stability, automated data acquisition software packages and the use of direct electron detectors [with improvements in detection quantum efficiency (DQE), information transfer, and dose fractionation], have revolutionized the Cryo-TEM research field [18, 31–34]. Not only have these technologies significantly advanced this field of research, they have also dramatically improved the ease of use of Cryo-TEM, particularly through automation, and lowered the threshold for researchers in adjacent fields to adopt this technology. There is truly a great level of excitement in the structural biology research community concerning the future of research using this method. Moreover, there is widespread belief that further technological improvements will undoubtedly lead to unexpected advances in this methodology. For instance, there is a great deal of hope that phase plates will dramatically enhance contrast in low dose Cryo-TEM images. In order to generate contrast, Cryo-TEM images must be collected under focus. While this generates contrast, it also introduces distortions and aberrations in the resulting images. In order to create three-dimensional structures of protein complexes, these aberrations must be corrected through image processing techniques. Using a phase plate not only substantially improves contrast, but also allows the images can be collected in-focus preventing aberrations from defocusing. These benefits are believed to dramatically enhance cryo-electron microscopy allowing researchers to study biological specimens traditionally considered intractable to analysis, such as small macromolecules. The cryo-electron microscopy scientific community widely believes phase plates will be a watershed technological improvement [35–38]. It is quite possible that the combination of phase plates and automated routines in electron detection and data management could allow for rapid, routine generation of atomic resolution structures answering biomedical questions previously intractable to study. This could have a dramatic effect on medicine and vaccine development. It is very conceivable that Cryo-TEM studies could become as common and standard as X-ray crystallography experiments within the next couple of years.
